# 2223. A comparative analysis of the detection of UTI pathogens via culture method and the Open Array-nanofluidic real time PCR method

**DOI:** 10.1093/ofid/ofac492.1842

**Published:** 2022-12-15

**Authors:** Pallavi Upadhyay, Fahida Surar, Geun Kim, Jay Reddy, Salika Shakir, Barbara D Alexander, Kimberly Hanson, Vijay Singh

**Affiliations:** HealthTrackRx, denton, Texas; HealthTrackRx, denton, Texas; HealthTrackRx, denton, Texas; HealthTrackRx, denton, Texas; ARUP Laboratories, Salt Lake City, Utah; Duke University School of Medicine, Durham, North Carolina; University of Utah, Salt Lake City, Utah; HealthTrackRx, denton, Texas

## Abstract

**Background:**

Urinary tract infections (UTI) are the second most prevalent microbial infection, impacting 150 million people globally every year. Traditionally, urine culture is considered the gold standard for UTI pathogen detection. However, molecular techniques, such as real time multiplex PCR tests targeting multiple pathogens, are becoming the alternative diagnostic tools for rapid detection of pathogens with the potential to provide quick diagnosis and enable targeted treatment. Due to the serious economic and healthcare utilization burden UTIs pose, early pathogen detection with a rapid turn-around-time to results has the potential to be instrumental for improving patient care and outcomes.

**Methods:**

A total of 300 deidentified patient samples that were previously tested via urine culture (ARUP laboratories, Utah) were subjected to real time PCR molecular testing employing the nanofluidic Open Array ® platform (HealthTrackRX, Texas). Statistical analyses were performed using R version 3.6.0.

**Results:**

Among 300 urine specimens studied, culture detected pathogens in 183 patient samples (61%), and 117 samples were deemed negative. Culture and PCR results demonstrated an overall agreement of 75.3% (n=226) with 59% positive (n=177) and 16.3% negative (n=49). Results for 24.7% samples (n=74) were discordant. A total of 2% (n=6) were culture positive and PCR negative, and 22.7% (n=68) were culture negative and PCR positive. Agreement between PCR and culture positive results was 0.97, 95%CI (0.94, 0.99) (177/183). Among the PCR positive samples (n=245), 32.7% were poly-microbial (n=80) and 67.3% were mono-microbial (n=165). In comparison, among culture positive samples (n=183), 33.3% (n=61) were polymicrobial and 65.6% (n=120) monomicrobial.

**Conclusion:**

Our study demonstrates that multiplex real time PCR-based detection of UTI bacterial pathogens has positive agreement with the traditional urine culture method. PCR based testing has the potential to deliver quick pathogen identification to enable targeted treatment options and medication adjustments. Further studies will correlate semi-quantitative cfu/mL culture values with semi-quantitative copies/mL PCR values to further refine result reporting and test performance.

**Disclosures:**

**Pallavi Upadhyay, PhD**, HealthTrackRx: Stocks/Bonds **Fahida Surar, B.S.**, HealthTrackRx: Salaried Employee **Geun Kim, M.S.**, HealthTrackRx: Salaried Employee **Jay Reddy, PhD**, HealthTrackRx: Stocks/Bonds **Barbara D. Alexander, MD**, Astellas: Advisor/Consultant|HealthtrackRx: Advisor/Consultant|HealthtrackRx: Grant/Research Support|Scynexis: Grant/Research Support|UpToDate: Advisor/Consultant **Kimberly Hanson, MD, MHS, FIDSA**, HealthTrackRx: Advisor/Consultant|HealthTrackRx: Clinical Advisory Board Member **Vijay Singh, PhD**, HealthTrackRx: Stocks/Bonds.

